# Highly Sensitive Electrochemical Determination of Alfatoxin B1 Using Quantum Dots-Assembled Amplification Labels

**DOI:** 10.3390/s150820648

**Published:** 2015-08-20

**Authors:** Xiaoqun Zeng, Huiju Gao, Daodong Pan, Yangying Sun, Jinxuan Cao, Zhen Wu, Zhenyu Pan

**Affiliations:** 1 Key Laboratory of Animal Protein Food Deep Processing Technology of Zhejiang, Ningbo University, Ningbo 315211, China; E-Mails: zengxiaoqun@nbu.edu.cn (X.Z.); huijugao@163.com (H.G.); sunyangying@nbu.edu.cn (Y.S.); caojinxuan@nbu.edu.cn (J.C.); woodsen@163.com (Z.W.); 2 Food Science & Nutrition Department, Nanjing Normal University, Nanjing 210097, China; 3 School of Electronic Engineering, Xidian University, Xi’an 710071, China; E-Mail: msnagisap@gmail.com

**Keywords:** quantum dots, layer-by-layer assembly, electrochemical immunoassay, AFB1 detection

## Abstract

A competitive electrochemical immunoassay for highly sensitive detection of AFB1 is demonstrated using layer-by-layer (LBL) assembled quantum dots (QDs) as labels. To investigate the effects of the higher sensitivity of square wave voltammetric stripping (SWV) and of the LBL technique on the proposed immunoassays, the proposed assay was compared to electrochemical (EC) and fluorescent immunoassays, which did not use LBL technology. Peanut samples were analyzed using the three immunoassays. The limits of detection (LODs) were 0.018, 0.046 and 0.212 ng/mL, respectively, while the sensitivities were 0.308, 1.011 and 4.594 ng/mL, respectively. The proposed electrochemical immunoassay displayed a significant improvement in sensitivity, thereby providing a simple and sensitive alternative strategy for determining AFB1 levels in peanut samples.

## 1. Introduction

The most toxic aflatoxin is aflatoxin B1 (AFB1), which is produced by the fungi *Aspergillus flavus* and *Aspergillus parasiticus* as a secondary metabolite. These types of fungi commonly infect agricultural crops such as oilseed and cereals. Aflatoxins are potent carcinogens, mutagens and teratogens, and the International Agency for Research on Cancer categorizes aflatoxins as group I carcinogens. Many countries regulate AFB1 due to its extreme toxicity and widespread occurrence in staple foods and feed [1]. The European Commission set the maximum level of AFB1 in groundnuts, nuts, dried fruits, and cereals at 2 ng/g [2]. Aflatoxins are occasionally detected in corn, peanuts, cottonseeds, nuts, spices, milk, cheese, and in a variety of other foods [3]. They are stable at high temperatures and consequently may resist cooking processes. Therefore, a worldwide effort should be triggered to develop analytical methods for the detection of aflatoxins.

To date, the commonly existing methods and strategies to determine aflatoxins are as follows: thin-layer chromatography (TLC), high-performance liquid chromatography (HPLC), overpressured-layer and enzyme-linked immunosorbent assays (ELISA), *etc.* TLC is a relatively economical method for the determination of aflatoxins with little equipment required, and is still commonly used in some developing countries where limited or no facilities exist for the monitoring of such toxins in food and feeds. Chromatographic analysis is widely used and accepted by governments for the detection of aflatoxins. HPLC (normal and reversed-phase) has been used in conjunction with UV absorption, fluorescence, mass spectrometry, and amperometric detection with different AFB1 cleanup procedures including solid-phase extraction (SPE), supercritical-fluid extraction (SFE), matrix solid-phase dispersion (MSPD), and immunoaffinity chromatography [4]. The application of cyclodextrins to the HPLC analysis of aflatoxins has also been developed. Recently, the use of a flow system with capillary electrophoresis has been reported. Although sensitive and accurate, most of the chromatographic methods are laborious, expensive, time-consuming, and unsuitable for analysis of a high number of samples. They also require sophisticated equipment and extensive AFB1 cleanup procedures. The development and application of biosensors for the detection of various aflatoxins has also been developed, but their use is limited by the regeneration of the receptor surface [5]. In light of the above considerations, there is still an urgent need for more rapid, sensitive systems capable of early detection of AFB1 in order to protect individuals exposed to high-risk environments and conditions.

In recent years, quantum dots (QDs) due to their unique characteristics, e.g., easy handling; excellent electrochemical properties; being ideal donors; and their ability to bioconjugate with specific biomolecules, while retaining their biologic activity, have led some to believe that QDs offer significant potential as labels for bioanalytical detection, including for use in immunoassays and immunosensors [6]. They also have been extensively studied in fluorescence resonance energy transfer (FRET)-based nanobiosensor for rapid and highly-sensitive detection and diagnosis of different kinds of molecules and diseases [7,8]. By detecting the metal content in QDs as the signal value, an analyte’s concentration can then be determined. Zhang *et al.* developed a novel direct competitive fluorescence-linked immunosorbent assay for AFB1 detection using QDs as the fluorescent label [9]. Despite its sensitivity and accuracy, the operation of fluorescence method is expensive, time-consuming and complex, which limits their application. Hence, electrochemical (EC) immunosensors with high sensitivity, simple and low-cost instrumentation and easy signal amplification have received increasing attention [10,11]. Masoomi *et al.* described a nonenzymatic sandwich-type electrochemical immunosensor for determination of AFB1 [12].

In this study, we reported a highly sensitive PbS QD-based AFB1 detection strategy using PbS QD layer-by-layer (LBL) assemblies as signal-amplification labels. The proposed strategy combined the EC method’s high heavy metal sensitivity with LBL-assembled PbS QDs’ dramatic signal amplification ability, which leads to subnanomolar sensitivity. We also investigated the effects of the EC and LBL approaches on immunoassay technology. Compared with EC and fluorescent immunoassays, the results for the EC immunoassay showed a lower AFB1 detection limit with good sensitivity.

## 2. Materials and Methods

### 2.1. Reagents and Materials

The antigen (AFB1-BSA), monoclonal anti-AFB1 antibody, N-hydroxysulfosuccinimid sodium salt (NHS), 1-Ethyl-3-(3-dimethyllaminopropyl) carbodiimide hydrochloride (EDC), casein, Tween 20, streptavidin (STV), and biotin were purchased from Sigma-Aldrich (Milwaukee, WI, USA). All other reagents were purchased from Sinopharm Chemical Reagent Co., Ltd (Shanghai, China). All chemical reagents were analytical grade, and ultrapure water was used throughout the experiment.

All the glass containers used in the EC analysis required an extra pre-cleaning treatment with 1% v/v sub-boiled nitric acid overnight and then a rinse with ultrapure water.

### 2.2. Apparatus

A constant temperature magnetic stirrer (Putian Instrument Manufacturing Co., Jiangsu, China) and a nitrogen protection device (made in our laboratory) were used in the QD nanoparticle synthesis. A transmission electron microscopic (TEM) image was obtained with a H600 TEM (Hitachi, Japan). The X-ray diffraction (XRD) characterization was performed using X-ray diffraction (Bruker, D8 Focus, Karlsruhe, Germany) with Cu-K radiation at room temperature. High Resolution TEM (HRTEM) was used to determine the structure of the produced PbS QDs nanoparticle synthesis.

A conventional three-electrode configuration was used for the EC measurements, with a glassy carbon electrode (3 mm in diameter, CH Instruments Inc., Shanghai, China) as the working electrode, a Ag/AgCl electrode as the reference electrode, and a platinum wire as the counter electrode. EC experiments were performed using a CHI10308 EC workstation (CH Instruments Inc., Shanghai, China).

### 2.3. Protocols

#### 2.3.1. Synthesis of PbS QDs

The PbS QDs used as fluorescent and elemental labels were synthesized as previously reported [13,14,15] using mercapto acetic acid (TGA) as a stabilizing agent, and lead nitrate and sodium sulfide as the raw materials.

#### 2.3.2. QDs Bioconjugation to Ab

The prepared PbS QDs bioconjugation with monoclonal anti-AFB1 antibody was achieved by the covalent crosslinking of the external carboxyl groups (introduced by the TGA molecules) coated on the QDs with the MAb amino groups. In brief, the prepared PbS QDs (1.0 mg) were suspended in 1 mL PBS buffer (0.01 M, pH 7.4) and then transferred the suspension into a centrifuge vial. After shaking for 30 min in the dark, 200 µL of MAb (1 mg/mL) was added to the vial and incubated for 2 h. Subsequently, the MAb-PbS composites were centrifuged at 838.5 g for 30 min to remove the supernatant and thrice washed with PBS buffer (0.01 M, pH 7.4) [16].

#### 2.3.3. Preparation of the LBL-Assembled MAb-(PbS)_2_

The preparation of the LBL-assembled MAb-(PbS)_2_ was prepared using the streptavidin-biotin system. To prepare the STV-PbS bioconjugate, 3.2 mg PbS QDs were suspended in 1 mL PBS buffer (0.01 M, pH 7.4) with 3.0 mg EDC and 3.0 mg NHS [17]. After shaking for 1 h, 0.20 mL STV (2 mg/mL) was added and incubated for 1 h. Finally, the STV-PbS QDs were centrifuged at 838.5 g and suspended in PBS (0.01 M, pH 7.4) for further use. The PbS-biotin bioconjugate was synthesized by mixing the biotin (1 µM) with 500 µL PbS QDs suspension (0.2 mg/mL) overnight at room temperature, followed by centrifugation. After the supernatant was removed, the pellet was resuspended in PBS buffer. The synthesized procedures of the MAb-PbS-STV were as follows: the prepared MAb-PbS suspension and 1.0 mg EDC were mixed in a centrifuge vial. The vial was shaken for 30 min in the dark and 200 µL STV (2 mg/mL) was added to the vial and incubated for 1 h. The MAb-PbS-STV composites were then centrifuged at 838.5 g for 30 min to remove the supernatant and resuspended in PBS buffer (0.01 M, pH 7.4) for further use [18].

The MAb-(PbS)_2_ assemblies were obtained as described in the literature [19]. In brief, the MAb-PbS-STV was transferred into a centrifuge vial. The PbS-biotin was added and incubated for 30 min. The composites were centrifuged at 838.5 g for 10 min to remove the supernatant, and then thrice washed with PBST (0.1% Tween 20, pH 7.4) to remove any non-specifically adsorbed PbS-biotin conjugate. The STV-PbS was added and incubated for 30 min, followed by centrifugation at 838.5 g for 10 min to remove the supernatant. The result was washed with PBST to obtain the MAb-(PbS)_2_ bioconjugate.

#### 2.3.4. Competitive Fluorescent Immunoassay Using MAb-PbS as Labels

Because AFB1 hapten only has a single binding site with the antibody, the immunoassay used a competitive format. The competition was established by the competitive binding between the analyte and the coating antigen (AFB1-BSA) previously immobilized in the microtiterplate on the limited MAb-PbS binding sites [20].

**Scheme 1:**
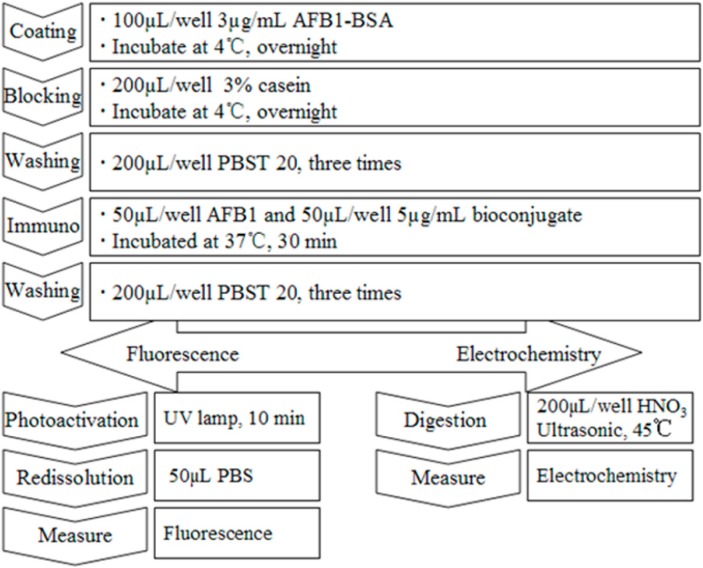
Quantum Dot-based immunoassay protocol.

For fluorescence detection, the microtiter plate was coated with 100 µL/well of 3 µg/mL AFB1-BSA and incubated overnight at 4 °C. Then, 200 µL of 3% casein solution was added as a blocking agent to every well and left overnight at 4 °C, to block the nonspecific binding sites as much as possible. The next step was to wash each well three times with 200 µL/well of a PBST buffer solution (0.1% Tween 20, pH 7.4). Then, 50 µL/well of AFB1 sample and 50 µL/well of prepared MAb-PbS solution (5 µg/mL) were added at the same time and incubated for 30 min at 37 °C, before the microtiter plates were washed three times. The microtiter plates were dried, followed by photoactivation for 10 min under a UV lamp. Finally, after a redissolution process with 50 µL of PBS, fluorescent measurements were taken using the excitation wavelengths (procedure described in Scheme 1).

The inhibition curves with the fluorescence intensity as the vertical coordinate and the AFB1 concentration as the abscissa were established. The inhibition curves responded to the four-parameter equation: *y* = [(A − D)/(1 + (*x*/C)^B^)] + D, where A is the maximum signal obtained; B is the slope of the curve; C is the sensitivity of the assay (IC_50_); and D is the background signal.

#### 2.3.5. Competitive EC Immunoassay Using MAb-PbS as Labels

Two hundred microliters/well of sub-boiled nitric acid was added to the dried microtiter plates to digest the MAb-PbS. The digestion was performed in an ultrasonic bath at a constant temperature of 45 °C for 30 min. The resulting solution was transferred into a 2 mL glass cell containing 1900 µL of acetate buffer (0.1 M, 10 ppm Hg^2+^, pH 4.5) for further EC measurement.

The square wave voltammetric stripping (SWV) detection involved a 2 min deposition at −1.1 V with a rotating disk electrode speed of 400 r/min, a potential from −0.8 to −0.2 V with a potential step of 4 mV, a frequency of 30 Hz and an amplitude of 25 mV [21]. 

The inhibition curves with the peak current value as the vertical coordinate and the AFB1 concentration as the abscissa were established. The inhibition curves responded to the four-parameter equation.

#### 2.3.6. Competitive EC Immunoassay Using MAb-(PbS)_2_ as Labels

The method was similar to the previous method, except that the competitive immunological reaction substituted MAb-(PbS)_2_ for MAb-PbS to add to the microtiter plate with the AFB1 sample. Other procedures and data processing were consistent with the former method.

#### 2.3.7. Sample Preparation

Peanut samples (20 g each) were purchased from retail supermarket in Ningbo, China. These samples were triturated in a blender, freeze-dried, and stored at 4 °C before analysis. The peanut samples were extracted according to previous methods with minor modification [22]. Spiked samples were obtained by mixing 1.0 g peanut samples with AFB1. 2 g sample was placed into a 10 mL tube, mixed with 4 mL acetonitrile, and ultrasonicated for 20 min at room temperature. Then, the mixture placed in a high-speed refrigerated centrifuge and centrifuged at 3354 g for 10 min. The extraction solutions were filtrated and concentrated under nitrogen. Finally, samples were quantified to 4 mL aqueous solution with 25% (m/v) NaCl for the further subsequent analysis.

## 3. Results and Discussion

The competitive immunoassay required a previous bioconjugation of a derivative of the monoclonal anti-AFB1 antibody to the colloidal water-soluble PbS for the subsequent fluorescence and EC detection. The bioconjugation consisted of the incubation of the antigen (AFB1-BSA) immobilized in the microtiter plates with the target AFB1, which came from standards or previously prepared samples competitively bound to the MAb-PbS.

### 3.1. Characterization of Water-Soluble PbS QDs With TGA as a Stabilizing Agent

**Figure 1:**
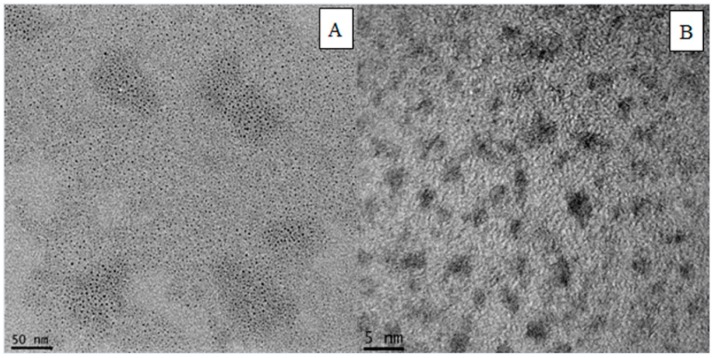
TEM (**A**) and HRTEM (**B**) images of PbS QDs.

We characterized TGA-capped PbS QDs using TEM and XRD to analyze their morphology and crystal structure. Figure 1 shows the typical TEM image of the synthesized PbS QDs. It was quite evident that these QDs were close to spherical with a high degree of monodispersity, while remaining well separated. They were about 3 to 5 nm.

**Figure 2:**
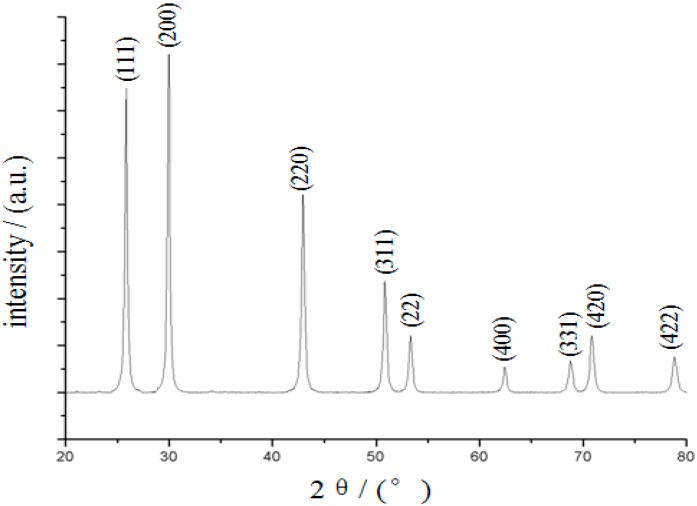
XRD patterns of the prepared PbS QDs.

Figure 2 shows a typical X-ray diffraction pattern of the powdered PbS QDs stabilized by TGA. All reflections matched well with the reference powder diffraction pattern of PbS. The positions of the diffraction peaks showed that the resulting TGA-stabilized PbS QDs had a cubic crystal structure. Because the prepared PbS QDs were so small, the half-band width was broader than the solid phase. Therefore, the experimental result from the XRD measurement agreed with the HRTEM images, confirming that the structure of the produced PbS QDs was face-centered cubic.

### 3.2. Optimization of Experimental Conditions

In order to obtain a better response of EC intensity using MAb-(PbS)_2_ as labels, the following parameters were optimized: (a) the pH of the electrolyte; (b) incubation time; and (c) incubation temperature. Respective data and Figures are given in the Supplemental Information. The following experimental conditions were found to give best results: (a) the pH of the electrolyte of 4.5; (b) an incubation time of 30 min; and (c) the incubation temperature of 45 °C.

### 3.3. Inhibition Curve of Three Competitive Immunoassays

A series of standard solutions with different AFB1 concentrations ranging from 0.01 to 1000 ng/mL were prepared (n = 3) following the procedure described previously, as shown in Scheme 1. After measurement, the obtained inhibition curves were plotted. From the inhibition curves, the analytical parameters were obtained using a four-parameter equation with Origin 8.0 software.

**Figure 3:**
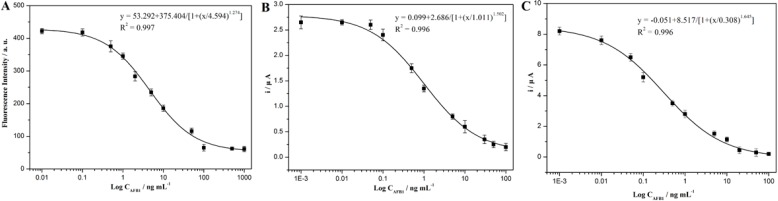
Inhibition curves for (**A**) fluorescence immunoassay using MAb-PbS as labels; (**B**) electrochemical immunoassay using MAb-PbS as labels; and (**C**) electrochemical immunoassay using MAb-(PbS)_2_ as labels.

The detection limit of the fluorescent immunoassay was defined as the concentration of the analyte that inhibited the measured analytical signal by 10% (IC_10_). Similarly, the sensitivity of the immunoassay was defined as IC_50_. Thee linear response range was the concentration interval between IC_20_ and IC_80_. According to Figure 3A, the sensitivity of the fluorescent immunoassay was 4.594 µg/L, the linear response range was 0.7–40 ng/mL, and the detection limit was 0.212 µg/L.

After EC measurements, the inhibition curve of the competitive EC immunoassay using MAb-PbS as labels was obtained. As shown in Figure 3B, the sensitivity of the EC immunoassay was 1.011 µg/L, the linear response range was 0.1–30 ng/mL, and the detection limit was 0.046 µg/L.

After EC measurements, the inhibition curve for the competitive EC immunoassay using MAb-(PbS)_2_ as labels was also obtained, as shown in Figure 3C, the sensitivity of the EC immunoassay was 0.308 µg/L, the linear response range was 0.04–15 ng/mL, and the detection limit was 0.018 µg/L.

### 3.4. Comparison of the Three Methods

Table 1 shows the analytical characteristics of the three methods. Under the same experimental conditions, the detection limit (IC_10_) obtained by competitive EC immunoassay using MAb-PbS as labels was 0.046 ng/mL, which was lower than obtained by competitive fluorescence immunoassay (0.308 ng/mL). The sensitivity (IC_50_) of the EC immunoassay using MAb-PbS as labels improved to 1.011 ng/mL of progesterone compared to only 4.594 ng/mL for the fluorescence immunoassay. Comparing the two methods, the EC detection method displayed higher sensitivity, a lower detection limit and a better detection of AFB1 than fluorescence detection. The inherently higher sensitivity of the SWV technique compared to fluorescence detection was probably due to the significant improvement in AFB1 detection caused by EC. It is worth stressing that this excellent SWV sensitivity to QD detection could be applied to any target biomolecule after modification of the immunoassay conditions. 

**Table 1:** Analytical characteristics of the inhibition curves

Parameter	Fluorescence (MAb-PbS)	EC (MAb-PbS)	EC (MAb-(PbS)_2_)
detection limit (ng/mL)	0.212	0.046	0.018
linear response range (ng/mL)	0.7–40	0.1–30	0.04–15
Sensitivity (ng/mL)	4.594	1.011	0.308
*R*^2^	0.997	0.996	0.996
Repeatability RSD (%)	9.3	5.7	12.3

It should be noted that the average relative standard deviation (RSD) obtained along the calibration curve using fluorescence was higher than using EC detection (9.3% and 5.7%, respectively). This finding suggests that the proposed method has satisfactory precision and acceptable reproducibility. The results of fluorescence detection highly depend on the quality control of the QD synthesis, which influenced the characteristics of the QDs, including their size, shape and surface morphology. Therefore, the fluorescence results may be improved by improving the quality of the QDs, but such an approach would require strict control of the experimental conditions, which would make it inconvenient to use for AFB1 detection. Moreover, the presence of other molecules in the detection system would change the surface properties of the QDs, thereby impacting the fluorescence intensity of the QDs and decreasing the assay’s accuracy. In addition, the stability was also investigated. After PbS QDs and MAb-(PbS)_2_ composites were stored at 4 °C for 28 days, they still maintained 94.5% and 95.6% of the initial responses, respectively. These findings indicated that the proposed immunoassay possesses good storage stability, thereby, increases its practical applicability.

When MAb-(PbS)_2_ were used as labels to replace MAb-PbS under the same experimental EC immunoassay conditions and procedures, a lower detection limit of progesterone down to 0.018 ng/mL was obtained. The sensitivity (IC_50_) of the assay improved to 0.266 ng/mL of AFB1, a value 19 times better than that previously obtained by fluorescence detection using MAb-PbS as labels, and three times better than that previously obtained by fluorescence detection using MAb-PbS as labels. The detection of such low AFB1 concentration levels was not even feasible using fluorescence detection because no coherent signals were obtained. Therefore, the use of a competitive EC immunoassay using MAb-(PbS)_2_ as labels exhibited excellent detection characteristics. Such attractive detection limits and sensitivity is primarily due to the inherent signal enhancement of QDs, the inherent high sensitivity of the SWV technique, and the substantial signal enhancement caused by the LBL-assembled QDs.

### 3.5. Application to Real Peanut Samples Analysis

To demonstrate the feasibility and real-world applicability of the proposed method, the competitive EC immunoassay using MAb-(PbS)_2_ as labels was also used to monitor AFB1 in real peanut samples. The real samples, which had no AFB1 detected, were spiked with AFB1 in concentrations from 0.1 to 10 ng/mL. Each concentration was measured five times, and the recovery rates were calculated. The recovery rates of the AFB1 standards in the real peanut samples were between 93% and 106% (Table 2). These desirable recovery rates definitely indicated the reliability of the proposed method for detection of AFB1 in real samples.

**Table 2:** The recovery of AFB1 in peanut samples (n = 5).

Sample Number	Additional Amount (µg/g)	Measured Results (µg/g)	Recoveries (%)	RSD (%, n = 5)
1	0.1	0.097	97	6.7
2	0.5	0.466	93	5.2
3	1	0.954	95	7.2
4	5	5.313	106	6.2
5	10	9.873	99	3.1

## 4. Conclusions

In this paper, we proposed a new immunoassay technology for the detection of AFB1 using LBL assembled QDs as labels. To investigate the signal amplification of the EC method, QDs and the LBL method, we compared the proposed method with EC and fluorescent immunoassays using MAb-PbS as signal labels. The EC immunoassay exhibited a lower AFB1 detection limit, which can be explained by two factors. First, the numerous ions released from QDs upon acid dissolution dramatically enhanced the current response, resulting in higher sensitivity than that obtained by fluorescent immunoassay. Second, sensitivity was further enhanced by inherently high sensitivity of the SWV technique due to the Pb^2+^ ions released from PbS QDs. Moreover, after combining with the LBL assembly, the QD-based EC immunoassay can produced even better performance. LBL assembly technology probably increased the number of Pb^2+^ ions released by the LBL-assembled QDs.

It should be noted that SWV is a technique for simultaneous high-resolution determination of many different components with high resolution. SWV can simultaneously detect a variety of metal ions in samples, including Pb^2+^, Cd^2+^ and Zn^2+^. Therefore, this approach can detect multiple analytes at the same time through different QDs (PbS, CdS and ZnS), which can contain different metal ions. This experiment provided the foundation for developing a method to simultaneously detect multiple analytes in the same sample based on different QDs.

## Supplementary Files

Supplementary File 1
