# Supplementary material for: Highly Sensitive Electrochemical Determination of Alfatoxin B1 Using Quantum Dots-Assembled Amplification Labels
Source: Sensors (Basel). 2015 Aug 20;15(8):20648–58. doi: 10.3390/s150820648 (PMC4570440; doi:10.3390/s150820648)
Supplement: Supplementary File 1 [file sensors-15-20648-s001.pdf]

OPEN ACCESS
sensors
ISSN 1424-8220
www.mdpi.com/journal/sensors

Supplementary Information

Highly Sensitive Electrochemical Determination of Alfatoxin B1 Using Quantum Dots-Assembled Amplification Labels. *Sensors* 2015, *15*, 20648-20658

Xiaoqun Zeng [1], Huiju Gao [1], Daodong Pan [1,2,*], Yangying Sun [1], Jinxuan Cao [1], Zhen Wu [1] and Zhenyu Pan [3]

[1] Key Laboratory of Animal Protein Food Deep Processing Technology of Zhejiang, Ningbo University, Ningbo 315211, China; E-Mails: zengxiaoqun@nbu.edu.cn (X.Z.); huijugao@163.com (H.G.); sunyangying@nbu.edu.cn (Y.S.); caojinxuan@nbu.edu.cn (J.C.); woodsen@163.com (Z.W.)

[2] Food Science & Nutrition Department, Nanjing Normal University, Nanjing 210097, China

[3] School of Electronic Engineering, Xidian University, Xi'an 710071, China; E-Mail: msnagisap@gmail.com

* Author to whom correspondence should be addressed; E-Mail: daodongpan@163.com; Tel.: +86-574-8760-8347.

The effect of pH of detection solution in the response of the competitive EC immunoassays using MAb-$(PbS)_2$ as labels was studied. For this purpose, the prepared bioconjugates were incubated in acetate buffer with various pH values at the constantly stirring state. The results of these experiments are shown in Figure S1A, as can be seen, with the increasing solution pH, the fluorescence intensity increased and achieved a maximum value at pH 4.5. According to these results, pH 4.5 acetate buffer, was chosen as an optimum pH for the EC measurements.

The effect of incubation time between prepared bioconjugates and AFB1 sample was also checked. The obtained results are shown in Figure S1B. According to this figure, both of EC intensity increased proportionally with the incubation time and reach a plateau at 30 min. Therefore, we chose an incubation time of 30 min in the experiments.

Additionally, since the reactivity of bioconjugates and and AFB1 sample is strongly affected by incubation temperature. The reaction was examined at 30, 35, 40, 45, 50, 55 and 60 °C. The results showed that the highest responses were obtained at 45 °C, as shown in Figure S1C, and then 45 °C was chosen as the favorable incubation temperature for determination.

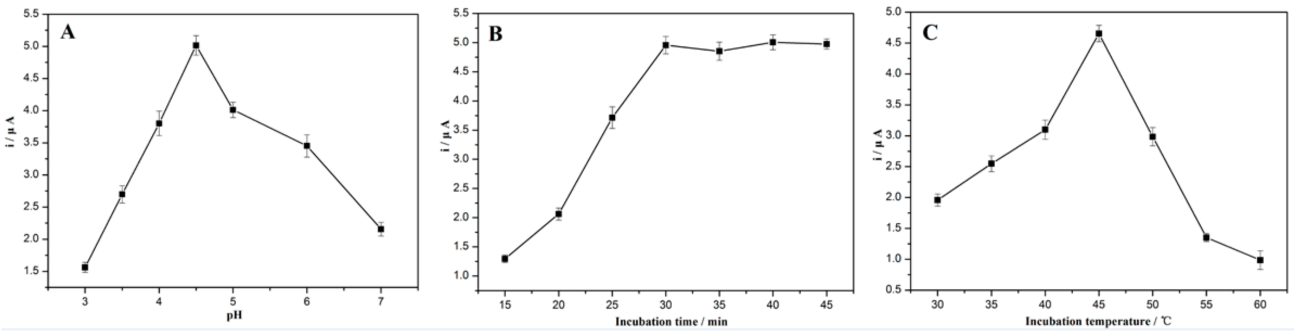

Figure S1. Dependences of EC intensity using MAb-$(PbS)_2$ as labels on pH (**A**), incubation time (**B**), incubation temperature (**C**) of the detection for measurement sample, when one parameter changed and the others were under their optimal conditions.

© 2015 by the authors; licensee MDPI, Basel, Switzerland. This article is an open access article distributed under the terms and conditions of the Creative Commons Attribution license (http://creativecommons.org/licenses/by/4.0/).
